# Effect of school health nurse-supported child-to-family strategy for Diabetic Retinopathy prevention in Bhimeshwor Municipality of Bagmati Province, Nepal: A quasi-experimental study

**DOI:** 10.1371/journal.pone.0357023

**Published:** 2026-09-02

**Authors:** Ramesh Shrestha, Chiranjivi Adhikari, Mohan Krishna Shrestha, Damaru Prasad Paneru, Amar Dawadi, Uma D.C

**Affiliations:** 1 School of Health and Allied Sciences, Pokhara University, Pokhara, Nepal; 2 Tilganga Institute of Ophthalmology, Kathmandu, Nepal; Concern Center for Rural Youth, Kathmandu, Nepal, NEPAL

## Abstract

**Introduction:**

Diabetic retinopathy (DR) is a leading cause of vision loss among individuals with diabetes, with prevention depending heavily on awareness and timely action. This study assesses the effectiveness of a school health nurse-supported child-to-family strategy for diabetic retinopathy prevention in Bhimeshwor Municipality, Nepal, focusing on students’ knowledge, family members’ knowledge and practices, and students’ retention in promoting health.

**Methods:**

A quasi-experimental pre-test–post-test control group design was conducted in purposively selected government schools with grades 7 and 8 that had school health nurse. One school was assigned as an intervention site and two schools as controls. A total of 140 students (70 intervention, 70 control) were selected using proportionate random sampling, each paired with a co-residing family member aged ≥30 years. The intervention included a six-week school nurse-led education program followed by student-led sessions at home. Quantitative data were collected using self-administered semi-structured questionnaires for students and face-to-face interviews for family members and analyzed using descriptive and non-parametric tests. Qualitative data was gathered through focus group discussions (one with students and one with family members) and a key informant interview with the school health nurse.

**Results:**

Post-intervention, students in the intervention group showed a significant increase in DR knowledge (median score: 0–16, *p* < 0.001), with large effect sizes (0.853) for knowledge and skills. Among family members, the intervention group demonstrated significant improvements in knowledge (median: 0–10, *p* < 0.001), attitude, perceived behavioral control, subjective norms, perceived severity, and self-efficacy (all *p* < 0.001), However, no significant change was observed in DR preventive practices, including regular eye check-ups, health tests (blood sugar and blood pressure), balanced diet and physical activity (*p* = 0.875). Qualitative findings supported the quantitative results, highlighting improved awareness, behavioral intentions, and family-level engagement.

**Conclusion:**

The intervention showed medium-to-high improvements in the experiential processes of diabetic-retinopathy prevention (knowledge, attitudes, subjective norms, perceived severity, perceived behavioral control and self-efficacy). However, behavioral change in actual preventive practices was not evident, likely due to the short intervention period. Approximately half of the students maintained high retention in sharing health information with their families. Strengthening experiential processes appears feasible, but further research with longer follow-up is needed to promote sustained behavioral change in diabetic-retinopathy preventive practices.

**Trial registration:**

The study trial was registered in the Australian New Zealand Clinical Trials Registry (ANZCTR). The trial registration number is **ACTRN12625000560493** on 02/06/2025.

## Introduction

Diabetes is a significant global health concern, affecting over 400 million people globally [1]. Its prevalence is anticipated to increase, with projections showing a rise among persons aged 20–79 from 6.9% in 2010 to 7.7% by 2030 [2]. This chronic illness is associated with multiple consequences, including cerebrovascular problems, renal failure, neuropathy, and retinopathy, which combined constitute a significant burden on healthcare systems [3].

Among these problems, diabetic retinopathy (DR) is a primary cause of vision impairment. Individuals with diabetes have a 25-fold increased risk of blindness than those without the illness [4]. Regular screenings, early medical interventions, and adequate control of associated risk factors are all necessary for effective DR management [1,5,6]. However, a lack of awareness remains a major barrier. For example, a study in Turkey found that 31% of diabetic patients lacked knowledge regarding the significance of eye care and were unaware of the potential impact of diabetes on their vision [7].

In Nepal, the prevalence of diabetic retinopathy (DR) was 23.8%, with vision-threatening DR accounting for 9.5% of cases. DR was most widespread (83.3%) among people who had lived with diabetes for more than 20 years [8]. Diabetes is a growing public health concern in Nepal, as indicated by the World Health Organization's focus on raising knowledge about its implications [6]. Preventing DR, a leading cause of blindness among people aged 20–64, necessitates coordinated community-wide measures. Studies show that timely treatments and appropriate eye care can prevent up to 90% of new DR cases [9]. However, there is still a gap in properly incorporating health awareness into existing educational systems.

Community-based strategies that encourage people to take preventive measures are critical in tackling such health issues. One potential option is the “child-to-family strategy,” which harnesses school-age children as change agents in their communities [10]. Children's ability to absorb and successfully spread new concepts makes them particularly positioned to impact family health behaviors [11,12]. This technique is consistent with the larger concept of “social vaccination,” which emphasizes the significance of awareness campaigns in promoting community health [13].

Schools are an ideal setting for providing health education, especially through innovative initiatives that involve children and their families. School health programs not only convey knowledge, but also develop an environment that supports beneficial health behaviors through collaboration among teachers, students, and healthcare professionals [14,15].

In 2018, the Government of Nepal implemented the “One School One Nurse” initiative, deploying trained nurses in schools to provide health education, first aid, screening, and referral support. This program aligns with the National Health Policy 2014, which emphasizes integrating health services into educational settings to promote student well-being, and underscores schools as platforms for community health promotion [16].

Preventing DR requires addressing knowledge, attitudes, and behavioral skills among students and their families. This study integrates selected constructs from three frameworks: the Theory of Planned Behavior (attitude, subjective norms, perceived behavioral control), the Health Belief Model (perceived severity), and Social Cognitive Theory (self-efficacy). Using these constructs allows the intervention to target cognitive, attitudinal, and behavioral determinants relevant to a child-to-family strategy. Guiding by this framework, this study aims to evaluate the effectiveness and feasibility of a school health nurse-supported child-to-family strategy for diabetic retinopathy prevention in Bhimeshwor Municipality, Bagmati Province, Nepal. Specifically, it assesses changes in students’ knowledge, family members’ knowledge and preventive practices, and students’ retention and continued engagement in promoting diabetic retinopathy prevention within their families.

## Materials and methods

### Study design and setting

The study was conducted in Bhimeshwor Municipality, Dolakha, Nepal, from January 26 to May 6, 2025, employing a quasi-experimental pre-test–post-test control group design with a mixed-methods approach.

### Study participants and sampling procedure

**Fig 1:**
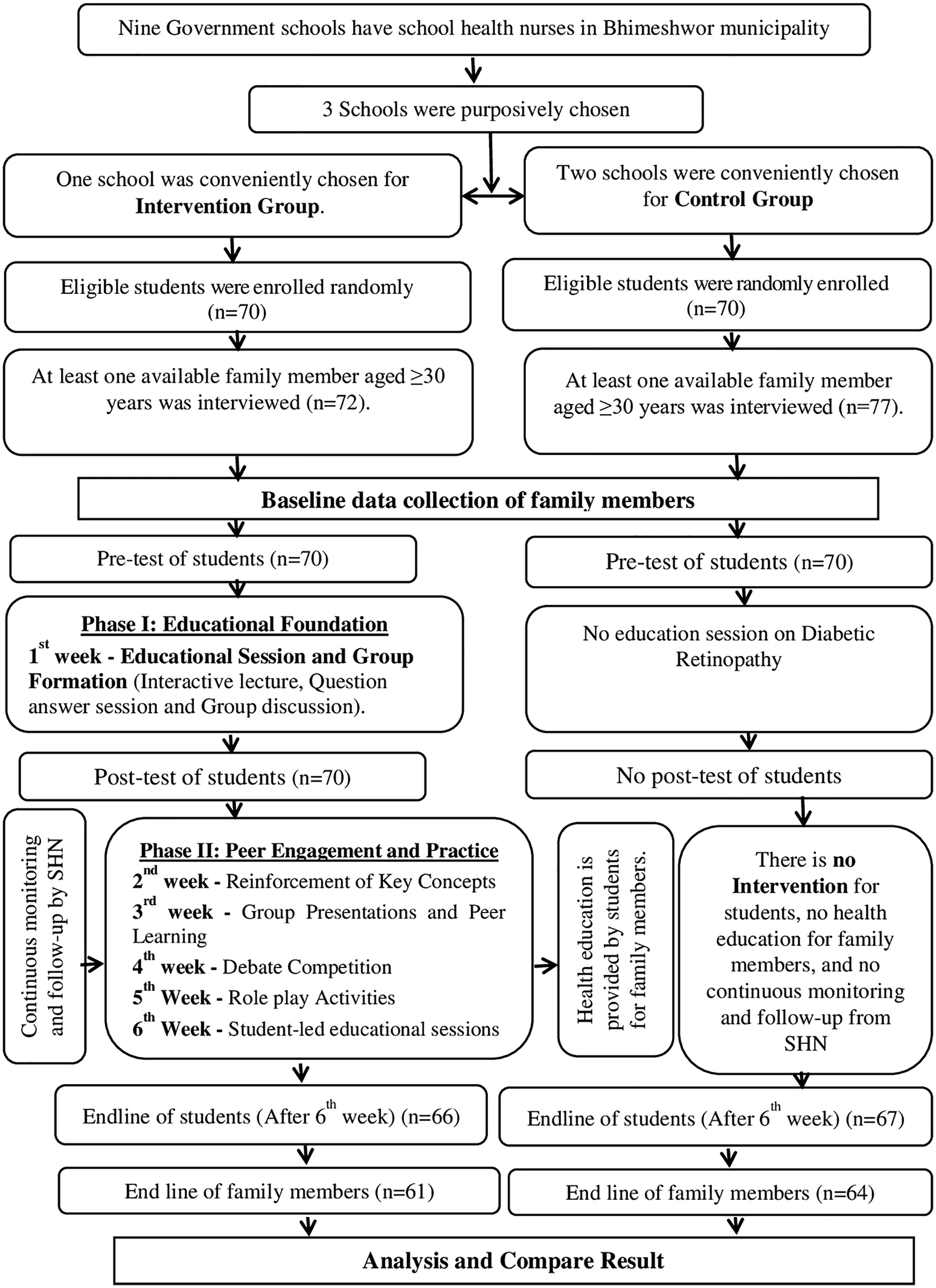
CONSORT participant flow diagram.

The study involved students from grades 7 and 8 and their co-residing family members aged 30 years and above. A total of 140 students were selected using proportionate random sampling, with 70 from the intervention school and 70 from two control schools. Proportionate random sampling was employed to ensure that the number of students selected from each school reflected the actual enrollment in grades 7 and 8, avoiding over or under representation. Within each school, students were assigned unique identification numbers, and participants were selected randomly from this list using a simple lottery method. Each selected student was paired with at least one eligible family member residing in the same household (Fig 1).

Schools were selected purposively from among government schools with assigned school health nurses, and the intervention and control schools were allocated based on convenience.

For the qualitative component, purposive sampling was employed to recruit participants for two focus group discussions – one with students and one with family members – and a key informant interview with a single school health nurse.

### Sample size calculation

The sample size was calculated based on a previous study in a Delhi school, where 19% of students demonstrated adequate knowledge of risk factors and lifestyle modifications [17]. Assuming an expected improvement to 40% post-intervention, with a 95% confidence level (Zα/2 = 1.96) and 80% power (Zβ = 0.84), the required sample size was determined using the standard formula for comparing two proportions.

N = [(Z_a/2_+ Z_β_)^2^.(p1(1-p1)+p2 (1-p2))]/(p1-p2)^2

The calculated sample size was 70 participants per group (intervention and control).

### Operational definitions

**School Health Nurse:** A School Health Nurse is a licensed healthcare professional who is assigned to a school or educational institution to provide health services, promote student well-being, and address physical, mental, and emotional health issues.

**Students (Children):** Students enrolled in grades 7 and 8 who live with at least one family member aged ≥30 years in the same household and share the same kitchen.

**Family member:** Individuals aged 30 years and above who live in the same household and share the same kitchen with the children.

**Retention:** The degree to which students continue delivering health education messages to their family members during the 42-day follow-up period. Students scoring below the median were classified as having low retention (inconsistent delivery), whereas those scoring at or above the median were classified as having high retention (sustained delivery throughout the 42 days).

### Data collection

Data was collected using a mixed-methods approach, integrating quantitative and qualitative techniques to comprehensively assess the impact of the intervention.

#### Quantitative data collection.

Quantitative data were collected at three time points: baseline (pre-test), immediate post-test (intervention group students only), and end line (after six weeks). For students, a self-administered structured questionnaire was used to evaluate four key domains: knowledge, skills, motivation, and retention. Knowledge was assessed using 18 multiple-choice questions. Skills were measured using seven items, incorporating binary (Yes/No) responses and a 5-point Likert scale ranging from 1 (“Not confident at all”) to 5 (“Extremely confident”), assessing students’ confidence in communicating health information to family members. Motivation was evaluated using three items that examined perceived importance, responsiveness to incentives, and the likelihood of applying acquired knowledge to influence family health behaviors.

Retention was assessed through daily logbooks maintained by students to track their involvement in family health education sessions. These logbooks were reviewed weekly by the school health nurse, who verified the completion of scheduled educational activities and monitored engagement as part of ongoing supervision and quality assurance.

For family members, data were collected through face-to-face interviews conducted by trained enumerators using a semi-structured questionnaire administered via Kobo Toolbox software on Android mobile devices. The questionnaire included socio-demographic items and scales informed by the Theory of Planned Behavior (TPB), Health Belief Model (HBM), and Social Cognitive Theory (SCT). Knowledge was assessed using 10 items, while attitude was measured with 9 items on a 4-point Likert scale. Preventive practices were evaluated with 5 items. Perceived behavioral control, subjective norms, self-efficacy, and perceived severity were each assessed using 5, 5, 5, and 8 items, respectively, all rated on 4-point Likert scales. Each interview lasted approximately 15–20 minutes.

The tools were developed by adapting items from previously validated instruments aligned with TPB, HBM, and SCT. Content validity was ensured through expert review by three public health specialists and one ophthalmic technician, followed by translation into Nepali, back-translation into English, and a pilot test among 14 students (10% of the study sample) and 10 family members (7.1% of the study sample) from a non-study site. Minor modifications were made to improve clarity and cultural appropriateness, and ease of administration.

Internal consistency was assessed using Cronbach’s alpha (α) based on the pilot test data from family members (n = 10). The values indicated excellent reliability: knowledge (α = 0.898), attitude (α = 0.984), perceived behavioral control (α = 0.983), subjective norms (α = 0.978), perceived severity (α = 0.965), and self-efficacy (α = 0.989).

#### Qualitative data collection.

Data were collected through two focus group discussions (FGDs)-one with students and one with family members- and a key informant interview (KII) with the school health nurse.

### Development of an intervention package

The intervention package was developed by the research team in consultation with public health and eye care experts. Educational content was adapted from the National Health Education, Information and Communication Centre (NHEICC) website and refined to ensure cultural relevance and feasibility for a school-based program. Development occurred within the context of this study at the participating schools, with attention to practical implementation by school health nurses. The final package included culturally tailored pamphlets, student logbooks, and session guides for a six-week school health nurse-led program, incorporating behavioral theory constructs.

### Intervention

#### Student intervention component.

The student intervention was conducted over six weeks and led by a school health nurse. It was divided into two phases: foundational learning and applied peer engagement.

### Phase I: Educational foundation

- **Week 1** – **Educational Session and Group Formation**Students from Grades 7 and 8 attended separate 3-hour interactive sessions, including lectures, group discussions, and Q&A activities. Students were divided into groups of 5–7 members, each with a group leader, and a student ambassador was selected per grade. Sessions focused on diabetes, diabetic retinopathy (DR) prevention, leadership, and health communication skills.

### Phase II: Peer Engagement and Practice

- **Week 2** – **Reinforcement of Key Concepts**Follow-up sessions were conducted to reinforce Week 1 content. Each session lasted 2 hours and was delivered separately to each grade.
- **Week 3** – **Group Presentations and Peer Learning**Each group gave a 15-minute presentation on DR prevention, followed by class-wide Q&A. Sessions lasted 3 hours per grade to encourage peer learning.
- **Week 4** – **Debate Competition:**Groups participated in 10-minute debates on DR topics. Health teachers served as judges, and prizes were awarded to the top three groups per grade. Sessions lasted 2 hours.
- **Week 5** – **Role-Play Activities:**Students performed 10-minute role-plays to demonstrate DR prevention concepts and communication strategies. Sessions lasted 2 hours per grade.
- **Week 6** – **Student-Led Educational Sessions:**Voluntary student-led 10-minute sessions were conducted, allowing students to review and teach key messages to their peers. Final sessions lasted 2 hours.

#### Family member intervention component.

Following each weekly session, students were instructed to share the acquired information with a co-residing family member aged 30 years or above daily. These children-led educational interactions occurred at home and were guided by the content provided in culturally appropriate pamphlets. Each student maintained a structured logbook to record the frequency, duration, and content of these sessions. Family members received information on diabetes and its complications, including the signs and symptoms of diabetic retinopathy, as well as key preventive behaviors such as blood sugar and blood pressure control, healthy dietary practices, regular physical activity, and the importance of routine eye examinations.

The school health nurse reviewed the logbooks weekly and conducted follow-up checks to monitor students’ engagement, provide feedback, and ensure the continuity and quality of the health education process.

### Outcomes

The primary outcome was the change in students’ knowledge of diabetic retinopathy after the intervention. Secondary outcomes included: (i) changes in knowledge, preventive practices, and perceptions among co-residing family members; (ii) student retention in health promotion activities; (iii) changes in perception and motivation among students; and (iv) the motivation of the school health nurse during the intervention.

### Data analysis

Quantitative data were processed and analyzed using SPSS Statistics version 25. Continuous variables with skewed distributions were summarized using medians and interquartile ranges (IQR), while categorical variables were described using frequencies and percentages. The primary outcome, students’ knowledge of DR, was evaluated using within-group comparisons (baseline vs post-test, post-test vs end line, baseline vs end line) with the Wilcoxon signed-rank test separately for the intervention and control groups. Between-group differences at baseline, post-test, and end line were assessed using the Mann–Whitney U test, and effect sizes (r = |Z|/√n) were calculated and interpreted as small (0.1–0.3), medium (0.3–0.5), or large (≥0.5) [18,19]. Secondary outcomes, including students’ skills and motivation on DR prevention, were analyzed with McNemar tests for within-group comparisons and chi-square tests for between-group comparisons. Retention of health promotion activities, measured by the frequency of student-family interactions, was summarized descriptively using frequencies and percentages. Family members’ knowledge, attitudes, and behavioral constructs were analyzed using Wilcoxon signed-rank tests for within-group changes and Mann–Whitney U tests for between-group comparisons, with effect sizes reported for all relevant outcomes. Categorical variables with small, expected counts, such as marital status, were analyzed using the Fisher’s exact test. A p-value < 0.05 was considered statistically significant for all analyses.

For qualitative data, thematic analysis was conducted manually by the primary researcher using an inductive approach. Transcripts were reviewed repeatedly to ensure familiarity with the data, and initial codes were developed iteratively. To ensure trustworthiness, an audit trail was maintained to document analytic decisions, peer consultation was conducted to review and refine emerging themes, and informal member checking during data collection helped validate interpretations.

### Benefits to participants and handling of possible risks

Participants gained awareness and knowledge about diabetes and diabetic retinopathy prevention through health education. The study involved no risk.

### Ethical considerations

Ethical clearance for the study was granted by the Institutional Review Committee (IRC) of Pokhara University (Ref No. 142/2081/82) on January 5, 2025. Before participation, students provided assent, and formal written consent was obtained from their parents and respective schools. For family members written informed consent was taken before study. The data collection process was carried out in the Nepali language to enhance comprehension. Participants retained the right to withdraw at any stage of the study. Anonymity was preserved through coded questionnaires, and all collected data were used exclusively for research purposes to ensure confidentiality.

## Results

### Baseline characteristics of the student participants

Table 1 presents the baseline characteristics of students in the intervention (n = 70) and control (n = 70) groups. The distribution by class was comparable, with 35.7% and 64.3% in grades 7 and 8 in the intervention group, and 41.4% and 58.6% in the control group, respectively (p = 0.487). A significantly higher proportion of students in the control group were aged ≤13 years (58.6%) compared to the intervention group (40.0%) (p = 0.028). Males constituted 71.4% of the intervention group, while only 45.7% in the control group were male (p = 0.002). Ethnicity distribution also differed significantly, with 57.1% Janajati and Dalit and 42.9% Brahmin and Chhetri in the intervention group, compared to 81.4% and 18.6%, respectively, in the control group (p = 0.002).

**Table 1:** Baseline characteristics of the students’ participants.

Variables	Intervention group (n = 70)		Control group (n = 70)		p-value
	n	%	n	%	
**Class**					
7	25	35.7	29	41.4	0.487^c^
8	45	64.3	41	58.6	
**Age Group**					
≤ 13 Years	28	40	41	58.6	0.028^c^
>13 Years	42	60	29	41.4	
Median (IQR)	14 (2)		13 (2)		
**Sex**					
Male	50	71.4	32	45.7	0.002^c^
Female	20	28.6	38	54.3	
**Ethnicity**					
Janajati and Dalit	40	57.1	57	81.4	0.002^C^
Brahmin and Chhetri	30	42.9	13	18.6	

^*c*^ *= Chi-square test; IQR = Interquartile Range; Statistical significance set at p < 0.05*

### Students’ knowledge on Diabetic Retinopathy prevention between groups over time

At baseline, there was no significant difference in knowledge scores between the intervention and control groups (median: 0 [IQR 0, 0]; p = 0.317). At the end of the study, the intervention group had significantly higher knowledge levels (median: 16 [IQR 15, 17]) than the control group (median: 0 [IQR 0, 0]; p < 0.001) (Table 2).

**Table 2:** Students’ knowledge on diabetic retinopathy prevention between groups over time.

Time Point	Group	n	Median (IQR)	p-value
**Baseline (Pretest)**	Intervention	70	0 (0,0)	0.317^m^
	Control	70	0 (0,0)	
**Posttest**	Intervention	70	14 (12,16)	–
**End line**	Intervention	66	16(15,17)	**<0.001** ^m^
	Control	67	0 (0,0)	

^*m*^ = *Mann-Whitney U test; IQR = Interquartile Range; Statistical significance set at p < 0.05; Post-test (immediate, same day); Endline (After 42 days)*

### Within-group comparison of students’ knowledge on Diabetic Retinopathy prevention over time

The intervention group showed significant improvements in knowledge scores from baseline to immediate post-test (p < 0.001, r = 0.858), post-test to end line (p < 0.001, r = 0.517), and baseline to end line (p < 0.001, r = 0.853). In contrast, the control group showed no significant change over time (baseline to end line: p = 0.285, r = 0.131) (Table 3).

**Table 3:** Within-group comparison of students’ knowledge on Diabetic Retinopathy prevention over time.

Group	Baseline-Post-test (B-P)	Effect Size (r)	Post-test-Endline (P-E)	Effect Size (r)	Baseline–End line (B-E)	Effect Size (r)
**Intervention**	**<0.001** ^w^	0.858	**<0.001** ^w^	0.517	**<0.001** ^w^	0.853
**Control**	**–**	–	–	–	<0.285^w^	0.131

^*w*^ *= Wilcoxon Signed-Rank Test; Statistical significance set at* p *< 0.05*

*B = Baseline (pretest); P = Post-test (immediate, same day); E = Endline (After 42 days);*

These findings are supported by qualitative responses from students, who reported gaining awareness and understanding of Diabetic Retinopathy through the intervention. One class 7 student stated, *“…I heard about Diabetic Retinopathy for the first time in this program itself, from the school health nurse”* (FGD-S1), while another class 7 student mentioned, *“….Now I understand that without timely checkups and treatment, it can lead to blindness”* (FGD-S3). Additionally, students could recall preventive measures, as reflected by (FGD-S7): *“….Regular eye check-ups, healthy eating habits, and reducing sugar intake can help prevent Diabetic Retinopathy.”* These narratives confirm the quantitative results, demonstrating that the intervention effectively enhanced students’ knowledge of DR.

### Between-group comparison of students’ skills on Diabetic Retinopathy prevention

At baseline, there was no statistically significant difference in diabetic retinopathy prevention skills between the intervention and control groups (p = 0.072). At the end, there was a significant difference between the two groups (p < 0.001) (Table 4).

**Table 4:** Between-group comparison of students’ skills on Diabetic Retinopathy prevention.

Time	Group	n	P-value
**Baseline**	Intervention	70	0.072^c^
	Control	70	
**End line**	Intervention	66	**<0.001** ^**c**^
	Control	67	

^*c*^ *= Chi-square test; Statistical significance set at p < 0.05*

The intervention group showed significant improvements in skills from baseline to post-test and end line (p < 0.001), with no change between post-test and end line (p = 0.500). The control group was assessed only at baseline and end line, showing a significant change between these time points (p < 0.001) (Table 5).

**Table 5:** Within-group comparison of students’ skills on Diabetic Retinopathy prevention.

Group	Baseline-Post-test (B-P)	Post-test-Endline (P-E)	Baseline–End line (B-E)
**Intervention**	**<0.001** ^m^	0.500^m^	**<0.001** ^m^
**Control**	–	–	**<0.001** ^m^

^*m*^ *= McNemar test; Statistical significance set at* p *< 0.05*

*B = Baseline (pretest); P = Post-test (immediate, same day); E = Endline (After 42 days);*

### Between-group comparison of students’ motivation on Diabetic Retinopathy prevention

At baseline, there was no significant difference in motivation between the intervention and control groups (p = 0.221). However, at the end, the intervention group had significantly greater motivation than the control group (p = 0.004) (Table 6).

**Table 6:** Between-group comparison of students’ motivation on Diabetic Retinopathy prevention.

Time	Group	n	P-value
**Baseline**	Intervention	70	0.221^c^
	Control	70	
**End line**	Intervention	66	**0.004** ^**c**^
	Control	67	

^*c*^ *= Chi-square test; Statistical significance set at p < 0.05*

### Within-group comparison of students’ motivation on Diabetic Retinopathy prevention

In the intervention group, no significant changes in motivation were observed between baseline, post-test, and end-line assessments (*p* > 0.05). Conversely, the control group showed a significant change in motivation between baseline and end line (*p* = 0.021) despite the absence of an intervention (Table 7).

**Table 7:** Within-group comparison of students’ motivation on Diabetic Retinopathy prevention.

Group	Baseline-Post-test (B-P)	Post-test-Endline (P-E)	Baseline–End line (B-E)
**Intervention**	1.000^m^	1.000^m^	0.754^m^
**Control**	**–**	–	**<0.021** ^m^

^*m*^ *= McNemar test; Statistical significance set at* p *< 0.05*

*B = Baseline (pretest); P = Post-test (immediate, same day); E = Endline (After 42 days);*

### Retention levels among intervention group students in sharing Diabetic Retinopathy education with family members

The frequency with which the 70 intervention students shared diabetic retinopathy prevention information with their family members was used to assess retention of health promotion activities. Based on the median sharing frequency of 25 sessions, students were divided into two groups: low retention (below 25) and high retention (25 or more). According to the findings, 51.4% of students demonstrated high retention by actively participating in the education-sharing activity, while 48.6% showed low retention (Table 8)

**Table 8:** Retention levels among intervention group students in sharing Diabetic Retinopathy education with family members.

S.N	Characteristics	Frequency	Percentage
1.	Low Retention	34	48.6
2.	High Retention	36	51.4
**Total**		**70**	**100**

*Median value = 25; Low retention = < 25, High retention = ≥25.*

### Baseline socio-demographic characteristics of family members in the intervention and control groups

Table 9 presents the baseline characteristics of family member participants in the intervention (n = 72) and control (n = 77) groups. The age distribution was similar across groups, with 56.9% of the intervention group and 55.8% of the control group aged ≥40 years (p = 0.892), and a median age of 40 years in both groups. The majority of participants were female, 72.2% in the intervention group and 71.8% in the control group (p = 0.914). Ethnicity differed significantly, with a higher proportion of Janajati and Dalit participants in the control group (89.6%) than in the intervention group (59.7%) (p < 0.001).

**Table 9:** Baseline socio-demographic characteristics of family members in the intervention and control groups.

Variables	Intervention group (n = 72)		Control group (n = 77)		p-value
	n	%	n	%	
**Age**					
<40 Years	31	43.1	34	44.2	0.892^C^
≥40 Years	41	56.9	43	55.8	
Median (IQR)	40 (7)		40 (10)		
**Sex**					
Female	52	72.2	55	71.8	0.914^C^
Male	20	27.8	22	28.6	
**Ethnicity**					
Janajati and Dalit	43	59.7	69	89.6	<0.001^C^
Brahmin and Chhetri	29	40.3	8	10.4	
**Marital Status**					
Married	68	94.4	76	98.7	0.198^F^
Divorced and a Widow	4	5.6	1	1.3	
**Educational Level**					
Illiterate	6	8.3	9	11.7	0.110^C^
Literate	25	34.7	19	24.7	
Basic education (Grade 1–8)	20	27.8	31	40.3	
Secondary education (Grade 9–12)	13	18.1	16	20.8	
Bachelor’s degree or above	8	11.1	2	2.6	
**Occupation**					
Agriculture	18	25	42	54.5	<0.001^C^
Business	20	27.8	3	3.9	
Government service	11	15.3	4	5.2	
Daily wage labor	8	11.1	5	6.5	
Private service	4	5.6	3	3.9	
Homemaker	11	15.3	20	26	
**Monthly Family Income (NPR)**					
<25000	32	44.4	59	76.6	<0.001^C^
≥25000	40	55.6	18	23.4	
Median (IQR)	25000 (23750)		15000 (10000)		

^*c*^ *= Chi-square test;* ^*F*^ *= Fisher’s Exact Test; IQR = Interquartile Range; Statistical significance set at p < 0.05*

Marital status and educational level distributions were not statistically different. Most participants were married (94.4% in intervention vs. 98.7% in control, p = 0.198), and a range of educational backgrounds was reported, with a higher proportion of participants holding a bachelor's degree or above in the intervention group (11.1% vs. 2.6%). Significant differences were observed in occupation and monthly family income. Agricultural work was more common in the control group (54.5% vs. 25%, p < 0.001), whereas business and government service were more frequent in the intervention group. Additionally, 76.6% of control group participants reported a monthly income below NPR 25,000 compared to 44.4% in the intervention group (p < 0.001).

### Comparison of knowledge, attitude, and behavioral constructs between intervention and control groups at baseline and end line with effect sizes

Between-group comparisons using the Mann–Whitney U test revealed significant improvements in key behavioral constructs among participants in the intervention group compared to the control group at end line. Median knowledge scores increased markedly in the intervention group (10 [IQR: 10–10]) versus no change in the control group (0 [IQR: 0–0], p < 0.001, r = 0.834). Similar significant differences favoring the intervention group were observed in attitude (27 [IQR: 27–28.75] vs. 0 [IQR: 0–0], p < 0.001, r = 0.761), perceived behavioral control (15 [IQR: 15–15] vs. 0 [IQR: 0–0], p < 0.001, r = 0.779), subjective norms (17.5 [IQR: 17.5–18] vs. 0 [IQR: 0–0], p < 0.001, r = 0.753), perceived severity (27 [IQR: 26–30] vs. 0 [IQR: 0–0], p < 0.001, r = 0.771), and self-efficacy (15 [IQR: 15–16.75] vs. 0 [IQR: 0–0], p < 0.001, r = 0.722). No significant difference was noted in preventive practices between the groups (p = 0.875, r = 0.014).

Within-group comparisons using the Wilcoxon signed-rank test indicated significant improvements from baseline to end line among participants in the intervention group across all constructs except preventive practices. Knowledge (p < 0.001, r = 0.872), attitude (p < 0.001, r = 0.837), perceived behavioral control (p < 0.001, r = 0.858), subjective norms (p < 0.001, r = 0.836), perceived severity (p < 0.001, r = 0.834), and self-efficacy (p < 0.001, r = 0.847) showed large effect sizes, indicating meaningful behavioral change. In contrast, changes in preventive practices were not statistically significant (p = 0.578, r = 0.072) (Table 10).

**Table 10:** Comparison of family members knowledge, attitude, and behavioral constructs between intervention and control groups at baseline and end line with effect sizes.

Group Variable	Intervention group (n = 72)		Control group (n = 77)		p-value	Effect Size (r)
	Median	IQR	Median	IQR		
**Knowledge**						
Baseline	0	(0,0)	0	(0,0)	0.200^m^	0.105
Endline	10	(10,10)	0	(0,0)	**<0.001** ^**m**^	0.834
**p-value**	**<0.001** ^**W**^		0.343^w^			
**Effect Size (r)**	0.872		0.118			
**Attitude**						
Baseline	0	(0,0)	0	(0,0)	0.151^m^	0.118
Endline	27	(27,28.75)	0	(0,0)	**<0.001** ^**m**^	0.761
**p-value**	**<0.001** ^**W**^		0.168^W^			
**Effect Size (r)**	0.837		0.172			
**Perceived Behavioral Control**						
Baseline	0	(0,0)	0	(0,0)	0.171^m^	0.112
Endline	15	(15,15)	0	(0,0)	**<0.001** ^**m**^	0.779
**p-value**	**<0.001** ^**W**^		0.078^W^			
**Effect Size (r)**	0.858		0.220			
**Subjective Norms**						
Baseline	0	(0,0)	0	(0,0)	0.202^m^	0.104
Endline	17.50	(17.50,18)	0	(0,0)	**<0.001** ^**m**^	0.753
**p-value**	**<0.001** ^**W**^		0.206^W^			
**Effect Size (r)**	0.836		0.158			
**Preventive Practices**						
Baseline	0	(0,4)	0	(0,0)	0.184^m^	0.109
Endline	0	(0,4)	2	(0,2)	0.875^m^	0.014
**p-value**	0.578^W^		0.118^W^			
**Effect Size (r)**	0.072		0.196			
**Perceived Severity**						
Baseline	0	(0,0)	0	(0,0)	0.181^m^	0.109
Endline	27	(26,30)	0	(0,0)	**<0.001** ^**m**^	0.771
**p-value**	**<0.001** ^**W**^		0.116^W^			
**Effect Size (r)**	0.834		0.197			
**Self-Efficacy**						
Baseline	0	(0,0)	0	(0,0)	0.179^m^	0.110
Endline	15	(15,16.75)	0	(0,0)	**<0.001** ^**m**^	0.722
**p-value**	**<0.001** ^**W**^		0.063^W^			
**Effect Size (r)**	0.847		0.233			

^*W*^ *= Wilcoxon matched-pairs signed-rank test;* ^*m*^ *= Mann-Whitney U test; IQR = Interquartile Range; Statistical significance set at p < 0.05*

The qualitative findings from family members provided context for these outcomes. At baseline, parents universally lacked awareness of diabetic retinopathy (DR):

*“…We had never even heard of Diabetic Retinopathy before.”* (FGD-FM1-6).

Post-intervention, the significant quantitative gains in knowledge (Table 10) aligned with parents’ reports of adopting preventive strategies:

*“…Since my child told us about it, we’ve reduced using too much oil and sugar in cooking.”* (FGD-FM1).

Improved attitudes (p < 0.001) correlated with families’ willingness to prioritize healthier habits:

*“…We’ve started eating more green vegetables and reduced sweets.”* (FGD-FM4).

Quantitative improvements in perceived severity (p < 0.001) reflected parents’ recognition of DR risks:

*“…Now we understand that without timely checkups and treatment, it can lead to blindness.”* (FGD-FM3).

The lack of improvement in preventive practices (p = 0.875) contrasted with parents’ efforts to adopt changes, as barriers such as ingrained habits emerged:

*“…Lack of time, difficulty in changing old habits, and initially not believing what the child said.”* (FGD-FM5).

Sustainability was supported by parents’ commitment to long-term behavioral adjustments:

*“…We’ve started buying more vegetables because of what our child taught us.”* (FGD-FM 6).

### Qualitative findings

The qualitative part of the study showed six main themes about participants’ experiences after the school-based program on preventing Diabetic Retinopathy (DR). These themes reflect improved knowledge, behavior changes, sharing of information, motivation, and the role of the school health nurse. Participants were coded to maintain confidentiality and facilitate data analysis. The following codes were used:

- FGD**: Focus Group Discussion**
- FM: Family Members
- **KII:** Key Informants Interview
- **S**: Students
- SHN: School Health Nurse

### Theme 1: Knowledge on Diabetic Retinopathy prevention

The findings reflected that there was no prior knowledge of Diabetic Retinopathy among students and family members. The program played crucial role in improving their understanding of its causes, symptoms, and prevention.

*• “…We had no idea about Diabetic Retinopathy.”* – (FGD-FM1–6)

*• “…I heard about Diabetic Retinopathy for the first time in this program itself.”* – (FGD-S1)

*• “…I didn’t know anything about it before, and now I know it’s a serious condition that can lead to vision loss and blindness.”-* (FGD-S8)

### Theme 2: Preventive practices on diabetic retinopathy

After the participation in program, families and students started adopting preventive practices such as reducing sugar intake, increasing vegetable consumption, and planning for regular health checkups.

*• “…My son now runs and exercises on our rooftop every morning. He learned this from the program. He also tells me and his mom to exercise, but we haven’t had time. We need to manage our time better.”* – (FGD-FM5)

*• “…We started putting less sugar in our tea at home.”* - (FGD-S5)

*• “…We’ve started eating more green vegetables and reduced sweets.”* – (FGD-FM4)

However, certain participants noted limited or delayed behavioral change even after participation in program

*• “…We haven’t made any major changes, but my daughter tells us not to eat too much sugar and oily food. We’ll gradually reduce it.”* – (FGD-FM 6)

*• “…My father said he knows alcohol affects the eyes and kidneys by causing diabetes, and that he should stop, though he admitted it’s hard to quit.”* – (FGD-S7)

### Theme 3: Perception of students and family members

The perception of both students and their family members shifted from ignorance to active awareness. Families appreciated the knowledge shared by children, seeing them as capable health educators.

*•“…Children could also teach us if guided well by the nurse and teachers.”* – (FGD-FM1)

*•“…They appreciated me sharing what I had learned and encouraged me to teach others.”* – (FGD-S2)

*•*“...My mother and father thanked me for talking about Diabetic Retinopathy and told me to teach it to our neighbors also.” – (FGD-S4)

### Theme 4: Behavior changes in students and family member

Participants explained behavioral changes included healthier eating, increased physical activity, and discussions about health within families.

*•“…Since my child told us about it, we’ve reduced using too much oil and sugar in cooking.”* (FGD-FM1)

*•“…I eat less sugar now and play during school break time.”* - (FGD-S1)

However, participants also found it difficult to consistently change habits.

*•“…I’ve reduced eating noodles and biscuits, though I still crave them and haven’t fully stopped. I do a bit of exercise in the morning on our house terrace and told my father to start running, though he hasn’t had time.”* – (FGD-S8)

*•“…We’ve started eating more green vegetables and reduced sweets. Haven’t started exercising yet but planning to.”* – (FGD-FM4)

### Theme 5: Motivation of students and family members

The students noted that they were motivated to share their knowledge and were even encouraged to seek health checkups.

- *“…I appreciated that my son asked me to go for a diabetes checkup. It made me happy to see him concerned about our health. I haven't gone yet due to time, but I will soon.”* - (FGD-FM5)
- *“…I told my father and mother. My father is a bit overweight, so I told him to go to the hospital for a blood test. He hasn’t managed to go yet due to time, but he said he would.”* - (FGD-S8)

Findings also reflected that family members were inspired, though others were constrained by time or habit.

*•“…Lack of time, difficulty in changing old habits, and initially not believing what the child said. But later, I realized it made sense*. - (FGD-*FM5)*

### Theme 6. Motivation of the school health nurse

The school health nurse was seen as an important and effective figure in delivering health information, motivating students, and indirectly influencing families.

*“…To be honest, I feel very proud to be working as a School Health Nurse. Even though it’s been only six months, I’ve realized how important our role is in shaping children's health habits from an early age. When I see students understanding health topics and sharing them with their families, it motivates me a lot. I feel like I’m not just doing a job but making a real difference in the community.” (KII-SHN)*

## Discussion

This quasi-experimental study assessed the effectiveness and feasibility of a school health nurse-supported child-to-family strategy for diabetic retinopathy (DR) prevention in Bhimeshwar Municipality, Dolakha. Among student participants, pre-test, immediate post-test, and end line assessments were conducted in the intervention group, while only pre-test and end line assessments were done in the control group to evaluate changes in knowledge and retention. For family members, baseline and end line assessments were carried out in both groups to assess changes in knowledge and practices related to diabetic retinopathy prevention. At baseline, significant differences were observed between the intervention and control groups in student characteristics such as age, sex, and ethnicity, with a higher proportion of older students, males, and Janajati/ Dalit ethnicity in the intervention group. Among family members, significant differences were found in ethnicity, occupation, and monthly family income, with a greater proportion of Janajati/Dalit ethnicity, agricultural occupation, and lower income in the control group. These baseline differences between the intervention and control groups highlight the need to be careful while interpreting the results, as some factors may have influenced the outcomes. The study design, with different assessment points for students and families, helped compare changes within each group. This approach supported the understanding of how the child-to-family strategy could improve awareness and practices related to diabetic retinopathy prevention at both the school and family levels.

The study demonstrated a significant increase in students’ knowledge of diabetic retinopathy (DR) prevention in the intervention group, with median scores rising from 0 at baseline to 16 at endline (p < 0.001), while the control group showed no change.

These findings support the effectiveness of nurse-led school interventions, consistent with studies from a systematic review, U.S.A., and Bangladesh showing improvements in student knowledge, behavior, and physical activity outcomes [20–22].

Qualitative data revealed that many participants learned about diabetic retinopathy for the first time through the program. The study shows that nurse-led school interventions could enhance student knowledge and support early prevention, emphasizing the need for targeted education in underserved communities.

The study showed a significant increase in diabetic retinopathy prevention knowledge among family members in the intervention group, with median scores rising from 0 to 10 (p < 0.001) and a large effect size (r) = 0.834). The control group showed no change.

These findings supported the effectiveness of child-to-parent health education strategies, consistent with studies from South India, Nepal, and Iran demonstrating improvements in family health knowledge and behaviors through student-led interventions [17,23,24].

Qualitative findings reinforced the quantitative results, showing that students effectively educated their families about diabetic retinopathy. Parents reported learning from their children about diabetes-related eye health risks, healthy eating, and lifestyle changes. This highlights successful knowledge transfer from students to family members.

Students showed notable improvements in communicating diabetes-related knowledge and promoting preventive behaviors within their households, acting as active transmitters of health information. Motivation, including perceived importance and readiness to act, did not change significantly. Most participants reported that extrinsic rewards, such as recognition or prizes, would increase their motivation, indicating that short-term interventions effectively enhance skills but may require extrinsic incentives or longer engagement to foster intrinsic motivation for health behavior change.

The study revealed a significant improvement in family members’ attitudes toward diabetic retinopathy prevention following a child-to-family educational intervention. Initially, both intervention and control groups had median attitude scores of 0 (IQR: 0–0). Post-intervention, the intervention group's median score increased to 27 (IQR: 27–28.75; p < 0.001), while the control group remained unchanged.

Although research specifically on student-led health education guided by the Theory of Planned Behavior (TPB) is limited, previous TPB-based interventions have shown positive effects in shaping attitudes toward diabetic retinopathy prevention [6,25]. The qualitative findings showed that participants became more aware of the importance of eye health, regular checkups, and healthy lifestyle practices. Participants expressed a stronger concern for preventing diabetic complications. These changes reflect the positive influence of student-led education on family health attitudes.

Quantitative analysis revealed a statistically significant improvement in perceived behavioral control (PBC) among family members, with median scores increasing from 0 to 15 (p < 0.001, effect size *r* = 0.858). This finding is consistent with prior research demonstrating that family-centered diabetes education enhances behavioral self-regulation and disease management, as evidenced by significant improvements in PBC following Theory of Planned Behavior (TPB)-based interventions [6,26].

Qualitative data supported these findings, with parents reporting healthier cooking habits-such as reduced oil and sugar use-prompted by their children's education. These accounts illustrate how child-driven learning could influence family behaviors and strengthen perceived control over diabetic retinopathy prevention, reinforcing the role of education in promoting effective diabetes management.

The intervention demonstrated a significant enhancement in subjective norms (SN) related to diabetic retinopathy (DR) prevention. Specifically, median SN scores increased markedly from 0 at baseline to 17.5 at endline (p < 0.001; effect size (r) = 0.836). This indicates a strengthened perception of social expectations regarding engagement in DR preventive behaviors. These results were consistent with previous research. A study conducted at the Diabetes Clinic in Arak reported higher mean SN scores post-intervention in the experimental group (21.11 ± 4.4) compared to the control group (16.22 ± 3.61) [6]. However, contrasting evidence from a cluster-randomized controlled trial [27] found no significant change in SN related to physical activity and healthy diet following the intervention. This discrepancy emphasized the context-specific nature of SN enhancement and highlighted the importance of culturally tailored, family-centered strategies for influencing health behaviors.

Qualitative findings from the present study further validated the quantitative outcomes. Students reported actively influencing their parents’ health behaviors by encouraging routine medical check-ups and advocating for healthier dietary choices. Parents also acknowledged modifying household behaviors-such as reducing oil and sugar use in cooking-based on their children's guidance. These accounts illustrate the crucial role of intergenerational communication and family dynamics in shaping health-related norms and practices. Together, qualitative and quantitative evidence reinforced the potential of educational interventions, particularly those leveraging child-to-family strategy, to strengthen subjective norms and promote sustainable DR preventive behaviors within households.

The current study observed a significant increase in perceived severity related to diabetic retinopathy (DR) prevention, with effect sizes of 0.834 for the intervention group and 0.197 for the control group. This improvement indicates a strengthened perception of the seriousness of DR among participants.

The current study’s findings were consistent with a study that examined the effects of an educational program based on the Health Belief Model (HBM) on adopting healthy behaviors among type 2 diabetic patients. That study found that the mean score of perceived severity in the intervention group increased significantly after 3 and 6 months, showing that the educational program effectively improved participants’ understanding of how serious diabetes and its complications can be [28].

The current study found significant improvement in self-efficacy among participants, consistent with previous research on diabetes management interventions. Meta-analysis found that empowerment-based interventions in adults with type 2 diabetes mellitus (T2DM) led to improvements in psychosocial self-efficacy, with a standardized mean difference (SMD) of 0.24 (95% CI: 0.10–0.37; P < 0.001) [29].

Similarly, a randomized controlled trial reported that a diabetes self-efficacy enhancing program significantly increased self-efficacy among older adults with T2DM, leading to improved self-care activities and reduced HbA1c levels [30]. These studies support the effectiveness of educational interventions in enhancing self-efficacy among individuals with diabetes, which is crucial for promoting self-management behaviors and preventing complications such as diabetic retinopathy.

The current study observed no statistically significant improvement in preventive practices for diabetic retinopathy (DR) following the intervention, with negligible effect sizes (r) = 0.072 for the intervention group and 0.196 for the control group. This contrasts with findings from other studies that demonstrated more substantial benefits from targeted interventions.

However, a meta-analysis reported that interventions targeting modifiable risk factors significantly reduced the risk of developing DR (OR = 0.60; 95% CI: 0.45–0.79) and its progression (OR = 0.62; 95% CI: 0.47–0.80; P < 0.001) [31]. Multifactorial interventions, particularly those with follow-up periods longer than five years, were more effective in reducing DR development and progression.

Similarly, the Finnish Diabetes Prevention Study reported a significant reduction in the occurrence of retinal microaneurysms among participants who underwent lifestyle interventions focusing on weight loss, healthy diet, and physical activity. The intervention group had a lower occurrence (24%) compared to the control group (38%; p = 0.029), suggesting that lifestyle modifications can positively impact early retinal changes associated with DR [32].

Qualitative insights from the current study provide context for these findings. Participants cited challenges such as “lack of time,” “difficulty in changing old habits,” and “initially not believing what the child said” as barriers to adopting preventive behaviors. These personal and social factors likely contributed to the limited behavioral changes observed quantitatively.

These studies suggested that more comprehensive and sustained interventions may be necessary to effect meaningful changes in preventive practices for DR. The limited impact observed in the current study may be attributed to factors such as the intervention's duration, intensity, or participant engagement levels.

In the intervention group, just over half of the students (51.4%) demonstrated high retention in sharing diabetic retinopathy education with their family members, while 48.6% showed low retention. This near-equal distribution suggested variability in student engagement and consistency in relaying health information at home, highlighting the need to explore factors that influence retention and information-sharing behaviors among students.

The findings of this study further support the Government of Nepal’s *‘One School One Nurse’* initiative by demonstrating the potential of school health nurses to extend health education beyond students to their families, thereby strengthening the policy’s vision of schools as platforms for community health promotion.

This study had several notable strengths. It introduced an innovative child-to-family education strategy that extended diabetic retinopathy awareness into households. The involvement of school health nurses ensured accurate and structured delivery of content. A mixed-methods design enriched the evaluation by capturing both quantitative outcomes and qualitative insights. Grounding the intervention in established behavioral theories enhanced its conceptual rigor. The use of culturally appropriate and pictorial materials improved accessibility for low-literacy participants. Family engagement fostered intergenerational learning and active participation. Moreover, the approach demonstrated feasibility and scalability in low-resource settings. However, the study's generalizability is limited by its single-site design and relatively small sample size. Schools were purposively selected from geographically co‑located and similar settings, which may restrict the wider applicability of the findings. Although baseline socio-demographic differences were observed between the intervention and control groups (age, sex, and ethnicity), these variables were examined during analysis and interpreted cautiously; residual confounding cannot be ruled out due to the quasi-experimental design. The reliance on self-reported measures of knowledge and preventive practices may have introduced reporting bias, potentially overestimating the true effect of the intervention. The short intervention duration may not have captured sustained behavioral changes. Finally, reliance on self-reported data and the absence of audio recordings in qualitative sessions may have introduced bias and affected data depth.

## Conclusion

The school health nurse-led intervention improved experiential processes of diabetic retinopathy prevention, including knowledge, attitudes, subjective norms, perceived severity, perceived behavioral control, and self-efficacy. Behavioral processes, specifically the actual adoption of preventive practices such as regular eye check-ups, dietary changes, and physical activity, showed limited improvement, likely due to the short duration of the intervention. Approximately half of the students maintained high retention in sharing health information with their families. These findings suggest that school-based programs can effectively enhance awareness and motivation, but longer-term implementation and reinforcement are needed to support sustained preventive behaviors. For policymakers, the study highlights the value of integrating school health nurse–led education into national programs while considering strategies to promote the translation of knowledge and motivation into consistent preventive actions.

## Supporting information

S1 File Parents baseline data. (CSV)

S2 File Student baseline (Pretest) data. (CSV)

S3 File Parents endline data. (CSV)

S4 File Student baseline (Posttest) data. (CSV)

S5 File Parents inferential data analysis. (CSV)

S6 File Student endline data. (CSV)

S7 File Student inferential data analysis. (CSV)

S8 File Retention. (CSV)

S9 File Study protocol. (PDF)

## Abbreviations

DR: Diabetic Retinopathy
HBM: Health Belief Model
PBC: Perceived Behavioral Control
SCT: Social Cognitive Theory
SHN: School Health Nurse
SPSS: Statistical Package for Social Sciences
TPB: Theory of Planned Behavior
