# Supplementary material for: Effect of school health nurse-supported child-to-family strategy for Diabetic Retinopathy prevention in Bhimeshwor Municipality of Bagmati Province, Nepal: A quasi-experimental study
Source: PLoS One. 2026 Sep 2;21(9):e0357023. doi: 10.1371/journal.pone.0357023 (PMC13537593; doi:10.1371/journal.pone.0357023)
Supplement: Supplementary file 1 [file pone.0357023.s009.pdf]

Study Protocol

Trial registered on ANZCTR

Registration number

ACTRN12625000560493

Ethics application status

Approved

Date submitted

22/01/2025

Date registered

2/06/2025

Date last updated

2/06/2025

Date data sharing statement initially provided

2/06/2025

Type of registration

Retrospectively registered

Titles & IDs

Public title

School Health Nurse Supported Child-to-Family Strategy for Diabetic Retinopathy Prevention in Bhimeshwor Municipality, Dolakha, Nepal

Scientific title

School Health Nurse Supported Child-to-Family Strategy for Diabetic Retinopathy Prevention in Bhimeshwor Municipality, Dolakha, Nepal - A Quasi-Experimental Study

Additional trial identifiers [1]

Nil Known

Universal Trial Number (UTN)

Trial acronym

Related trial records

Health condition

Health condition(s) studied:

Diabetes

Diabetic Retinopathy

Condition category

Condition code

Metabolic and Endocrine

Diabetes

Eye

Diseases / disorders of the eye

Intervention/exposure

Study type

Interventional

Description of the intervention or exposure

This study aims to assess the effectiveness and feasibility of a school health nurse-supported child-to-family strategy to improve knowledge and preventive practices related to diabetic retinopathy (DR) among students and their families in Bhimeshwar Municipality, Dolakha, Nepal. The intervention specifically targets gaps in knowledge and preventive practices regarding DR, particularly among family members aged 30 and above.

Materials

For Students:

• Health Pamphlets: Culturally relevant content on diabetes, DR prevention, lifestyle changes, and eye check-ups.

• Pictorial Materials: Visual aids.

• Intervention Toolkit: Printed and digital resources to support learning.

For Family Members:

• Health Pamphlets: Simple, culturally appropriate content on diabetes, DR prevention, lifestyle changes, and regular eye check-ups.

Procedures:

The school that receives the intervention will be the first school that gives written consent and meets the inclusion criteria. All schools will be informed about this process before giving consent. This method fits with our study design and helps us start the intervention smoothly and fairly.

The intervention will involve school health nurses delivering educational sessions to students in Grades 7 and 8. Afterward, students will educate their family members, particularly those aged 30 and above, about DR prevention practices.

Timeline and Frequency:

The intervention will be conducted over six weeks, divided into two phases:

Pre-Intervention Assessment:

• **Baseline Assessment of Parents:**

Conduct baseline assessments of parents to evaluate their knowledge and practices regarding diabetes and diabetic retinopathy before the intervention.

• **Educational Sessions for Intervention Students:**

Hold separate educational sessions for Grades 7 and 8 students in the intervention group. Topics will cover diabetes and diabetic retinopathy prevention, healthy lifestyle habits, eye check-ups, and developing communication and leadership skills to enable students to share health messages with their family members.

• **Pre-tests and Post-tests for Intervention Students:**

Administer pre-tests before the educational sessions and post-tests after the sessions to assess knowledge gain in the intervention group.

• **Pre-test for Control Group Students:**

Conduct a pre-test for the control group students to serve as a baseline for comparison of results in the later stages of the study.

Post-Intervention Assessments:

Six weeks after the initial phase, conduct post-intervention assessments for both parents (Intervention and Control groups) and students (Intervention and Control groups) to evaluate the impact of the educational sessions and the overall intervention.

Sessions Frequency and Duration:

1. Student Education Sessions for Intervention Group:

Week 1: Introduction and Group Formation

Group Formation: Students will be divided into groups of 5-7 members, with one leader per group. An ambassador will be selected from each grade.

Pre-test: A pre-test will be conducted before the educational sessions.

Educational Sessions: Separate sessions are offered for Grades 7 and 8, covering DR prevention, communication strategies, and leadership skills, with each session lasting 3 hours for each grade.

Examples of lifestyle changes emphasized to address and prevent diabetic retinopathy (DR):

1. Maintaining Healthy Blood Sugar Levels

Example: Consistently monitoring and managing blood glucose levels through diet, exercise, and medication can prevent or slow the progression of diabetic retinopathy.

2. Healthy Diet

Example: Eating a balanced diet rich in fruits, vegetables, whole grains, lean proteins, and healthy fats helps in maintaining optimal blood sugar levels and supporting overall eye health.

3. Regular Physical Activity

Example: Engaging in regular exercise (such as walking, swimming, or cycling) helps regulate blood sugar levels, reduce body weight, and improve circulation, which can protect against the development of retinopathy.

4. Blood Pressure Control

Example: Keeping blood pressure within a healthy range through a combination of diet, exercise, and medication reduces the risk of developing diabetic retinopathy, as high blood pressure can worsen eye damage.

5. Managing Cholesterol Levels

Example: Monitoring and controlling cholesterol levels through diet, exercise, and medication helps protect the blood vessels in the eyes, which are at risk due to diabetes-related vascular damage.

6. Regular Eye Exams

Example: Scheduling regular eye exams with an ophthalmologist to detect early signs of diabetic retinopathy. Early detection can lead to timely treatment and prevent vision loss.

7. Smoking Cessation

Example: Quitting smoking helps improve circulation and overall health, reducing the risk of complications like diabetic retinopathy and other cardiovascular diseases.

8. Stress Management

Example: Practicing stress management techniques such as meditation, yoga, or mindfulness helps in managing blood sugar levels and reduces the risk of developing complications related to diabetes.

Post-test: A post-test will be administered after the session.

Pamphlets and Logbook: Students will receive pamphlets and a daily tracking logbook to monitor their engagement. These logbooks will be reviewed weekly by the school health nurse, who will also collect and verify that the weekly educational activities were completed by students with their family members.

Support and Troubleshooting: If any challenges arise while providing education to family members, they will be discussed within the team or with the school health nurse.

Week 2: Reinforcement of Key Concepts

The school health nurse will conduct follow-up educational sessions for both grades to reinforce the concepts covered in Week 1, with each session lasting 2 hours for each grade.

Week 3: Group Presentations and Peer Learning

Each group will present their learnings for 15 minutes, followed by a question-and-answer

session to promote peer learning. The total session will last 3 hours for each grade.

Week 4: Debate Competition
A debate on diabetic retinopathy (DR) prevention will be held for 10 minutes per group between groups from each grade. Prizes will be awarded to the top three groups from each grade, with health teachers serving as judges. The total session time will be 2 hours.

Week 5: Role-Play Activities
Students will perform a 10-minute role-play on DR prevention, conducted separately for each grade to demonstrate their understanding interactively. The session will last for 2 hours.

Week 6: Student-Led Educational Sessions and Final Assessments
Voluntarily, students will lead educational sessions for their classmates for 10 minutes to reinforce key concepts. The session will last for 2 hours.
Post-intervention assessments will be conducted 42 days after the first educational session in both the intervention and control groups to evaluate knowledge retention among students and changes in knowledge.

2. Family Education Sessions:
Daily Family Sessions:
Students will conduct daily educational sessions with their family members aged 30 and above (5-20 minutes).

Logbook Recording: Students will record the daily activities in a logbook. If a session is not conducted, students will document the reason for not providing the educational message. This helps track consistency and provides insight into any barriers faced.

Content Delivery Method:
Face-to-face sessions will be delivered by the students to their family members using printed health pamphlets as support materials.

Key Personnel:

School Health Nurse: A trained healthcare professional responsible for delivering educational sessions to students and supervising the intervention.

Students: Grades 7 and 8 will be trained by the school health nurse to communicate DR prevention effectively to their families.

Research Team: Monitors the intervention and collects data under the guidance of the principal investigator.

Location:

Intervention Settings: The intervention will take place in selected schools in Bhimeshwar Municipality, Dolakha.

Family Settings: Family-based education will occur in students' homes by students.

Intervention Modifications:

Engagement Challenges: If challenges arise in participant engagement, materials may be modified with additional visual aids or more relatable examples, based on feedback from students and families.

Adherence and Fidelity:

Supervision: Close monitoring of educational sessions by the school health nurse and research team.

Student Monitoring: Students will report on their interactions with family members and any difficulties encountered.

Strategies for Maintaining Fidelity:

Ongoing support for the school health nurse.

Regular feedback loops to assess the intervention’s effectiveness.

Refresher sessions if necessary to maintain fidelity.

Allocation to Intervention/Control Group:

Study Design: The intervention will be implemented in one school, and two schools will

serve as control schools. These schools have been purposively selected based on the availability of a school health nurse and the number of students.

Control Group: The control schools will continue with their standard practices without the intervention, allowing for comparison with the intervention school.

Allocation Process: The intervention school will be assessed first, with assessments conducted before and after the intervention. The control schools will receive the intervention after the first set of schools have completed the intervention phase.

Planned vs. Actual Adherence:

Planned Adherence: Ensuring students and families receive and understand the educational material within the established timeline.

Actual Adherence: Monitored through session attendance, intervention completion, and feedback from students and families.

Intervention code [1]

Prevention

Description of the comparator or control

The study will include one intervention school and two control schools. Two schools were selected for the control group because each has fewer students, and combining them allows for a 1:1 ratio of participants between the intervention and control groups.

Students in the control group will not receive diabetic retinopathy prevention sessions. Instead, they will attend general health education sessions on unrelated topics, delivered by the school nurse. These sessions are designed to keep students engaged without influencing outcomes related to diabetic retinopathy.

Both groups will undergo baseline and post-intervention assessments at the same time points to evaluate differences in knowledge and preventive practices. This design will help assess the effectiveness of the School Health Nurse-Supported Child-to-Family Strategy for Diabetic Retinopathy Prevention.

Control group

Active

Outcomes

Primary outcome [1]

Knowledge change on Diabetes and Diabetic Retinopathy among students

Assessment method [1]

For assessing the primary outcome, a validated knowledge inventory will be used, specifically the Diabetes and Diabetic Retinopathy Knowledge Questionnaire. This tool is well-established and has been validated to measure knowledge related to diabetic retinopathy.

Timepoint [1]

Baseline and 6 weeks following end of education sessions.

Secondary outcome [1]

Knowledge change on Diabetic Retinopathy among family members age 30 and above

Assessment method [1]

For assessing the secondary outcome, a validated knowledge score will be used, derived from the Diabetic Retinopathy Knowledge and Attitude Questionnaire. This score will be based on the responses to the questionnaire, which has been validated for use in evaluating knowledge and attitudes related to diabetic retinopathy.

Timepoint [1]

Baseline and 6 weeks following end of baseline

Secondary outcome [2]

Preventive practice change in family members age 30 and above

Assessment method [2]

Preventive practices will be assessed using a questionnaire developed specifically for this study. The questionnaire will focus on assessing the preventive practices related to diabetic retinopathy, including lifestyle modifications, eye care, and regular check-ups. A pre-test will be conducted to evaluate the validity of the questionnaire before its use in the study. The questionnaire will be designed to capture both knowledge and behaviors, ensuring a comprehensive assessment of preventive practices among participants.

Timepoint [2]

Baseline and 6 weeks following end of baseline

Secondary outcome [3]

Student retention in health promotion activities will be assessed to measure ongoing engagement and participation throughout the intervention, helping evaluate its effectiveness in maintaining involvement.

Assessment method [3]

Retention rate data will be collected through a weekly logbook provided to students to record their daily activities for up to 6 weeks (42 days). The logbook will include the student's name, grade, code number, topics discussed with family members, total time spent, and the time of day the discussion took place. If a session is not conducted, students will be required to note the reason for not sharing the educational message. This logbook will help track student involvement in health promotion activities and monitor retention throughout the intervention period.

Timepoint [3]

Following the initial educational session, post-intervention assessments will be conducted at the end of 6 weeks. Data will be collected at specific timepoints: Baseline: Prior to the intervention, baseline assessments will be conducted for both students (intervention and control) and their family members (intervention and control). Post-Intervention: After 6 weeks, post-intervention assessments will be conducted to evaluate changes in knowledge among students in both the intervention and control groups, as well as changes in knowledge and preventive practices among their family members (intervention and control). Follow-Up: In the intervention group, retention data will be collected weekly using a logbook throughout the 6-week intervention period to track students' involvement in health promotion activities.

Secondary outcome [4]

Perception, behavior changes, and motivation among family members and students.

Assessment method [4]

Focus group discussion among family members and students.

Timepoint [4]

At the end of the study.

Secondary outcome [5]

Motivation among School Health Nurses

Assessment method [5]

Key Informant Interview

Timepoint [5]

At the end of study

Eligibility

Key inclusion criteria

Inclusion criteria

For students:

- Aged 10 years and above
- Enrollment in grades 7 or 8 in the selected school
- Parents' consent to participate
- Student's assent to participate in the study
- Living with family members of age 30 and above.

For family members:

- Aged 30 years and above
- Reside in the same household as the selected student, sharing the same kitchen
- Available for participation in the intervention and follow-up assessments during the study period

Minimum age

10 Years

Maximum age

80 Years

Sex

Both males and females

Healthy volunteers

Yes

Key exclusion criteria

For students:

• Students who are differently abled (blind, deaf, or non-verbal.)

• Students whose family members do not live with them.

For family members:

• Family members were diagnosed with diabetic retinopathy.

• Family members are unable to participate in both the baseline and end-line surveys.

• Family members who are differently abled (blind, deaf, or non-verbal.)

Study design

Purpose of the study

Prevention

Allocation to intervention

Non-randomised trial

Procedure for enrolling a subject and allocating the treatment (allocation concealment procedures)

Methods used to generate the sequence in which subjects will be randomised (sequence generation)

Masking / blinding

Open (masking not used)

Who is / are masked / blinded?

Intervention assignment

Other

Other design features

Quasi experimental (Intervention and control group purposively selected)

The selection process for this study will be carried out as follows: Schools will be purposively selected based on specific inclusion criteria, including the presence of a school health nurse. Within each selected school, students will be chosen using simple random

sampling, with the number of students selected from grades 7 and 8 being proportional to the total number of students in these grades. Additionally, the parents of the selected students, aged 30 and above, who are available during the data collection period, will be interviewed.

Phase

Not Applicable

Type of endpoint/s

Statistical methods / analysis

Recruitment

Recruitment status

Completed

Date of first participant enrolment

Anticipated

Actual

26/01/2025

Date of last participant enrolment

Anticipated

Actual

5/02/2025

Date of last data collection

Anticipated

Actual

6/05/2025

Sample size

Target

70

Accrual to date

Final

70

Recruitment outside Australia

**Country [1]**
Nepal
State/province [1]
Bagmati

Funding & Sponsors

**Funding source category [1]**
University
**Name [1]**
School of Health and Allied Sciences, Pokhara University
**Country [1]**
Nepal
**Primary sponsor type**
Individual
**Name**
Ramesh Shrestha, School of Health and Allied Sciences, Pokhara University
**Country**
Nepal
**Secondary sponsor category [1]**
None
**Name [1]**
**Country [1]**

Ethics approval

**Ethics application status**
Approved
**Ethics committee name [1]**
Pokhara University Institutional Review Committee
**Ethics committee address [1]**
**Ethics committee country [1]**
Nepal

**Date submitted for ethics approval [1]**	
29/12/2024	
**Approval date [1]**	
05/01/2025	
**Ethics approval number [1]**	

Summary

Brief summary

Trial website

Public notes

Contacts

Principal investigator

Name

Mr Ramesh Shrestha

Address

School of Health and Allied Sciences, Pokhara University, Pokhara Metropolitan City-30 Kaski, Nepal

Country

Nepal

Phone

+977 9843697989

Email

rameshshrestha0826@gmail.com

Contact person for public queries

Name

Ramesh Shrestha

Address

School of Health and Allied Sciences, Pokhara University, Pokhara Metropolitan City-30 Kaski, Nepal

Country

Nepal

Phone

+977 9843697989

Email

rameshshrestha0826@gmail.com

Contact person for scientific queries

Name

Ramesh Shrestha

Address

School of Health and Allied Sciences, Pokhara University, Pokhara Metropolitan City-30 Kaski, Nepal

Country

Nepal

Phone

+977 9843697989

Email

rameshshrestha0826@gmail.com

Data sharing statement

Will the study consider sharing individual participant data?

Yes

Will there be any conditions when requesting access to individual participant data?

Persons/groups eligible to request access:

• Researchers

• Individual researcher and organization.

Conditions for requesting access:

• -

What individual participant data might be shared?

• De-identified participant data, excluding any information that could reveal individual identities, will be shared upon request. Data will be made available for research purposes with appropriate citation and approval from the Principal Investigator (PI). The shared data will include [specify: all collected data, data related to published results, or primary outcome data only, as applicable].

What types of analyses could be done with individual participant data?

• Systematic reviews and meta-analyses

• For primary and secondary analysis

When can requests for individual participant data be made (start and end dates)?

From:

At the end of the study

To:

No end date

Where can requests to access individual participant data be made, or data be obtained directly?

• On request to PI and corresponding author. email: rameshshrestha0826@gmail.com

Are there extra considerations when requesting access to individual participant data?

No

Study Protocol Link:

https://anzctr.org.au/Trial/Registration/TrialReview.aspx?id=389028&isReview=true
